# The big five personality traits, perfectionism and their association with mental health among UK students on professional degree programmes

**DOI:** 10.1186/s40359-020-00423-3

**Published:** 2020-06-02

**Authors:** Elisa G. Lewis, Jacqueline M. Cardwell

**Affiliations:** 1 Present address: Division of Psychology, London South Bank University, 103 Borough Road, London, SE1 0AA UK; 2 Pathobiology and Population Sciences, Royal Veterinary College, Hatfield, Hertfordshire AL9 7TA UK

**Keywords:** Students, University, Personality, Perfectionism, Mental health, Healthcare professions

## Abstract

**Background:**

In view of heightened rates of suicide and evidence of poor mental health among healthcare occupational groups, such as veterinarians, doctors, pharmacists and dentists, there has been increasing focus on the students aiming for careers in these fields. It is often proposed that a high proportion of these students may possess personality traits which render them vulnerable to mental ill-health.

**Aim:**

To explore the relationship between the big five personality traits, perfectionism and mental health in UK students undertaking undergraduate degrees in veterinary medicine, medicine, pharmacy, dentistry and law.

**Methods:**

A total of 1744 students studying veterinary medicine, medicine, dentistry, pharmacy and law in the UK completed an online questionnaire, which collected data on the big five personality traits (NEO-FFI), perfectionism (Frost Multidimensional Perfectionism Scale), wellbeing (Warwick-Edinburgh Mental Well-being Scale), psychological distress (General Health Questionnaire-12), depression (Beck Depression Inventory-II) and suicidal ideation and attempts.

**Results:**

Veterinary, medical and dentistry students were significantly more agreeable than law students, while veterinary students had the lowest perfectionism scores of the five groups studied. High levels of neuroticism and low conscientiousness were predictive of increased mental ill-health in each of the student populations.

**Conclusions:**

The study highlights that the prevailing anecdotal view of professional students possessing maladaptive personality traits that negatively impact on their mental health may be misplaced.

## Background

Previous research has suggested that healthcare students, such as those training for medical, veterinary, pharmacy and dentistry degrees, may be at increased risk of mental ill-health [1–3]. Studies involving law students suggest that this population also experiences heightened levels of distress [4–6]. A recent direct comparison of the mental health and wellbeing of veterinary, medical, pharmacy, dentistry and law students in the UK found that law students experienced the poorest mental health of all these groups, while veterinary and medical students reported comparatively better mental health [7].

It is often proposed that the reported poor mental health and elevated rates of suicide identified in the veterinary [8, 9] and other healthcare professions [10–12] are rooted in a predominance of maladaptive personality traits in people attracted to these occupations. However, while it is recognised that there are associations between mental health and personality traits, there is, to date, no consistent evidence that particular traits are over-represented in specific student populations.

Research has demonstrated a number of associations between mental health and the personality dimensions described by the five-factor model of personality, which encompasses conscientiousness, agreeableness, neuroticism, extraversion and openness to experience. For example, a higher level of neuroticism is a recognised risk-factor for depression and suicidal behaviour [13, 14], while extraversion is associated with more positive mental health [15].

One US study found that medical students were within average ranges relative to age-matched general population norms for neuroticism, agreeableness and conscientiousness, but had significantly higher levels of extraversion and openness [16]. Furthermore, Meit et al. [17], using the 16 Personality Factor Questionnaire [18], reported that US medical students were warmer and more emotionally stable than the general public. Together, these findings suggest that the medical students studied were not excessively afflicted with maladaptive personality traits, but in fact may have higher levels of the traits associated with positive mental health, personal growth, leadership, assertiveness and adaptability than the general population [16, 17].

Associations between conscientiousness and academic success have been consistently identified in a number of studies involving medical students [19–21]. Conscientiousness has also been associated with more positive mental health and adaptive coping skills [22, 23]. A 12-year longitudinal study of UK medical students found that lower levels of conscientiousness and extraversion, and higher levels of neuroticism, were predictive of greater stress and burnout in doctors [24], while a six-year longitudinal study in Norway [25] identified that a combination of high neuroticism, high conscientiousness and low extraversion predicted medical school stress. These studies point to a complex relationship between conscientiousness and psychological distress, which may be mediated by other personality traits and particular contexts.

It is also often suggested that healthcare students have a tendency towards high levels of perfectionism [26–28]. Students are selected for professional degree courses on the basis of consistent academic excellence and high personal standards, which must be maintained throughout university. Therefore, it is plausible to suggest that the very traits which allow students to gain entry onto the degree course could also be those which increase a tendency towards perfectionism. Perfectionism is a multifaceted trait, which may be both adaptive and maladaptive [29–31]. Maladaptive perfectionism, such as socially-prescribed perfectionism (a perceived need to meet the often unrealistic standards and expectations of others [32]) is associated with increased risk of depression, anxiety and suicide [18, 32–34] as well as increased reluctance to seek help for psychological distress [35]. On the other hand, adaptive perfectionism is associated with achievement-striving and setting of personal goals, without excessive self-criticism [29, 36]. It is also positively correlated with positive affect, greater agency and conscientiousness [26, 37].

Among US medical students, significantly lower levels of maladaptive perfectionism have been reported in comparison with arts students [26]. While maladaptive perfectionism was associated with depression, neuroticism, hopelessness and suicidal ideation, adaptive perfectionism was significantly associated with conscientiousness, self-reported academic expectations and achievements [26]. A comparison of perfectionism in medical, dental, nursing and pharmacy students found that pharmacy students reported the highest levels of socially-prescribed perfectionism [27]. Those students who scored highly on the perfectionism scale were at a significantly greater risk of psychological distress than others. However, the healthcare students in this study did not report more perfectionism than other student groups and scored significantly lower overall than the general student population on socially-prescribed perfectionism. Again, these findings do not support the suggestion that healthcare students are more perfectionistic than their peers.

It is often suggested anecdotally that the veterinary profession may attract individuals with personality traits that render them susceptible to mental ill-health, and that this could contribute to the heightened suicide risk evidenced in this population [38, 39]. In particular, a popular opinion is that veterinary students or graduates are problematically perfectionistic high achievers. One US study sought to use a combination of validated questionnaires to determine whether veterinary students possess similar characteristics, including perfectionism, to those found among elite, high achieving populations, such as ‘high achiever’ college students and professional athletes [28]. Although the authors concluded that veterinary students possessed a strong fear of failure and elevated levels of anxiety, these conclusions were not supported by the data presented. In fact, the data suggested generally lower mean scores for anxiety and the maladaptive dimensions of perfectionism in veterinary students compared with other groups. However, there was no statistical assessment of the magnitude or significance of the reported variation.

To date there are no published UK-based studies directly comparing the distribution of personality traits among veterinary students with those of other similar student populations. As such, it cannot be ascertained whether there is a disproportionate distribution of maladaptive personality traits among veterinary students compared with other healthcare students. Furthermore, healthcare students as a group could differ from other student populations owing to the increased emotional and empathy demands of their degree course. For this reason a group of law students was included in the present research. Law students were selected because arguably they are subject to similar stressors of long working hours and heavy workloads and their degree course is also career focused. Law as a profession, however, differs from healthcare in that it emphasises objectivity and may encourage a more dispassionate approach.

The aim of this study was to explore relationships between the big five personality traits, perfectionism and mental health in UK students on professional degree programmes (veterinary medicine, medicine, pharmacy, dentistry and law). Objectives were (i) to use the 5-factor and perfectionism assessments to describe the distribution of personality traits and perfectionism across these five groups and (ii) to investigate associations between personality traits or perfectionism and mental health in these groups.

## Methods

### Participants

Recruitment for the study has been described previously [7]. Briefly, undergraduate students of veterinary medicine, medicine, pharmacy, dentistry and law, in every year of study, at seven English universities were invited to participate. The entry requirement at each university was at least AAA at A-Level. Ethical approval was sought from and granted by all participating institutions (RVC Ethics Code: 2013 0073H). An online tool SurveyMonkey® (www.surveymonkey.com) was used to administer the questionnaire.

### Questionnaire

The questionnaire comprised previously published and validated measures:

- Demographic questions (age, gender, UK citizenship, year of study and previous degrees).
- A measure of the five broad domains of personality (conscientiousness, agreeableness, neuroticism, openness to experience and extraversion) using the 60-item NEO-FFI [40]. There are 12 items for each domain measured on a five-point Likert scale from ‘strongly disagree’ to ‘strongly agree’. Participants are asked to choose the response that best represents their opinion. Each item is scored from 0 to 4, yielding a possible maximum score for each item of 48, with higher scores indicating greater levels of the measured trait. Items comprised statements similar to the following; ‘I rarely feel fearful or anxious’ and ‘I waste a lot of time before settling down to work’.
- Assessment of perfectionism using the Frost Multidimensional Perfectionism Scale (FMPS) [41]. The questionnaire comprises 35 items, which are answered using 5-point responses ranging from ‘strongly disagree’ to ‘strongly agree’. Scores may be obtained for each of the six subscales that reflect domains of perfectionism, namely Concern over Mistakes, Personal Standards, (the perception of high) Parental Expectations, (the perception of high) Parental Criticism, Doubts about Actions, and Organisation. Following guidelines by Frost et al. (1990), an overall perfectionism score was calculated by summing all the subscales with the exception of Organisation. Although Organisation is implicated in the more positive features of perfectionism and for this reason it is included as a separate factor in its own right, it is only weakly correlated with overall perfectionism and the other subscales [41]. Possible overall perfectionism scores range from 29 to 145.
- Assessment of wellbeing using the Warwick-Edinburgh Mental Well-being Scale [42]. The scale comprises fourteen positively-phrased 5-point Likert items scored from 1 (none of the time) to 5 (all of the time), providing a minimum score of 14 and a maximum score of 70. A higher score indicates higher levels of wellbeing.
- Assessment of non-psychotic psychological distress using the 12-item General Health Questionnaire [43]. The questionnaire includes 12 four-point Likert items to assess respondents’ mental health over the past few weeks in comparison with their usual state. Items are scored as 0,1,2,3 and summed to give an overall scale from 0 (least distressed) to 36 (most distressed).
- Assessment of depression using the 21-item Beck Depression Inventory-II [44]. Each item includes four statements scored from 0 to 3. Respondents select one statement from each item which best describes the way they have been feeling during the past two weeks. Scores are summed to give a range from 0 to 63. Higher scores indicate a greater severity of depression.
- Assessment of suicidal ideation and suicide attempts using two questions from the Adult Psychiatric Morbidity in England survey [45]. The questions were ‘Have you ever thought of taking your life, even if you would not really do it?’ and ‘Have you ever made an attempt to take your life, by taking an overdose of tablets or in some other way?’ Response options were ‘Yes, most recently in the last 12 months’, ‘Yes, most recently more than 12 months ago’ and ‘No’.

### Data analysis

Data were analysed using SPSS Statistics for Windows, version 21.0 (2012, Armonk, NY: IBM Corp). NEO-FFI raw mean scores, standard deviations and t-scores are reported for each of the five factors. T-scores were calculated using the formula t = 50 + 10*z, where z = (raw score – mean)/standard deviation. Mean values and standard deviations of the FMPS are reported. Raw mean scores for both the NEO-FFI and the FMPS were compared by student group using one-way ANCOVAs, followed by Bonferroni post-hoc tests, with age and gender as covariates to adjust for any confounding.

One-sample *t*-tests were used to compare mean NEO-FFI scores with published figures from approximately age-matched UK nursing students [46]. Data were only included if they were published within the last 20 years and involved UK, non-clinical populations. At the time of writing, no published data met these criteria for comparisons with the FMPS scores.

Outcome variables for multiple linear regressions were wellbeing (WEMWBS score), psychological distress (GHQ-12 score) and depression (BDI-II score). The outcome variable for the logistic regression was presence or absence of previous suicide attempts. Models were built using a manual forward stepwise approach, meaning that variables of interest, based on our research question were entered first, and the model was checked after each step to determine which variables should be retained. The variables age and gender (male [baseline], female) were entered first, followed by student population (veterinary [baseline], medical, pharmacy, dentistry and law). Age and gender variables were removed if there was no evidence of confounding. Neuroticism, extraversion, conscientiousness, agreeableness, openness to experience and perfectionism were then entered in turn, in order of univariable significance. Finally, year of study (1st [baseline], 2nd, 3rd, 4th and final), previous degree (yes [baseline], no), and UK [baseline] or non-UK Citizen were examined. Variables were retained in the model if significantly associated with the dependent variable at the 5% level. Age and gender were tested again at the end of model-building for evidence of confounding. Interaction terms between all final model main effects were examined and subject to a Bonferroni correction with a significance threshold of 0.005. Data were assessed for multicollinearity by checking that tolerance statistics were < 10 and variance inflation factor was > 0.2.

## Results

### Respondents

Demographic details have been summarised previously [7]. There were 1744 respondents in total. The mean age of participants was 22 years (SD = 3.42) and 80% of the total respondents were female, reflecting gender and age distributions in the study populations. Most participants (82%) were UK citizens who did not hold a previous degree.

### Personality

Scores from each of the five NEO-FFI domains are summarised overall and by student group in Table 1. After adjusting for age and gender, there was significant variation in openness to experience (*p* <  0.001) and agreeableness (*p* <  0.001) across groups. Pharmacy students scored significantly lower on openness to experience than veterinary (*p* = 0.01), medical (*p* <  0.001) and law (*p* <  0.001) students. Dentistry students also scored significantly lower on this domain than the medical (*p* = 0.001) and law students (*p* = 0.001). Veterinary (*p* < 0.001), medical (*p* < 0.001) and dentistry (*p* < 0.001) students obtained significantly higher agreeableness scores than the law students.

**Table 1:** Mean scores, standard deviations and T scores on each of the five NEO-FFI domains, by population and overall

	Population											
	Veterinary		Medical		Pharmacy		Dentistry		Law		Overall	
	Mean (SD) 95% CI	Mean t-score (SD)	Mean (SD) 95% CI	Mean t-score (SD)	Mean (SD) 95% CI	Mean t-score (SD)	Mean (SD) 95% CI	Mean t-score (SD)	Mean (SD) 95% CI	Mean t-score (SD)	Mean (SD) 95% CI	Mean t-score (SD)
Neuroticism	24.75 (8.84) 24.10–25.39 (*N* = 728)	50.05 (9.93)	24.06 (8.86) 22.86–25.26 (*N* = 212)	49.28 (9.96)	25.55 (8.69) 24.09–27.02 (*N* = 137)	50.96 (9.77)	23.85 (8.41) 22.49–25.21 (*N* = 149)	49.05 (9.45)	24.98 (9.79) 23.68–26.28 (*N* = 221)	50.32 (11.00)	24.67 (8.94) 24.21–25.13 (*N* = 1447)	49.96 (10.04)
Extraversion	27.58 (6.64) 27.10–28.06 (*N* = 726)	50.26 (9.77)	28.11 (6.76) 27.19–29.02 (*N* = 212)	51.05 (9.95)	26.19 (6.66) 25.05–27.32 (*N* = 134)	48.22 (9.79)	28.03 (7.18) 26.87–29.20 (*N* = 149)	50.93 (10.56)	26.69 (7.25) 25.73–27.65 (*N* = 221)	48.96 (10.67)	27.44 (6.83) 27.09–27.79 (*N* = 1442)	50.06 (10.05)
Openness	28.89 (6.04)* 28.45–29.33 (*N* = 726)	49.98 (9.90)	30.15 (6.40)‡ 29.28–31.02 (*N* = 211)	52.05 (10.49)	26.79 (5.19)*‡ ^†^ 25.91–27.68 (*N* = 135)	46.54 (8.51)	27.33 (6.19)‡ ^†^ 26.33–28.33 (*N* = 149)	47.42 (10.14)	29.89 (6.14) ^†^ 29.08–30.70 (*N* = 222)	51.61 (10.06)	28.87 (6.13) 28.56–29.19 (*N* = 1443)	49.95 (10.05)
Agreeableness	31.24 (5.99)* 30.81–31.68 (*N* = 723)	50.39 (9.51)	32.36 (6.35) ‡ 31.50–33.22 (*N* = 211)	52.16 (10.07)	30.22 (5.92) 29.22–31.22 (*N* = 136)	48.76 (9.38)	31.99 (6.19) ^†^ 30.99–32.99 (*N* = 149)	51.57 (9.81)	29.01 (6.82)*‡^†^ 28.10–29.91 (*N* = 221)	46.84 (10.82)	31.05 (6.27) 30.72–31.37 (*N* = 1440)	50.07 (9.95)
Conscientiousness	30.22 (7.14) 29.70–30.74 (*N* = 727)	49.88 (9.65)	30.62 (7.39) 29.62–31.62 (*N* = 211)	50.43 (9.99)	29.99 (7.59) 28.70–31.27 (*N* = 136)	49.57 (10.26)	31.58 (7.30) 30.40–32.76 (*N* = 149)	51.72 (9.87)	29.63 (8.13) 28.56–30.71 (*N* = 221)	49.10 (10.10)	30.30 (7.40) 29.92–30.69 (*N* = 1444)	50.00 (10.01)

Scores marked with *, †, and ‡ represent significantly different pairwise comparisons

Table 2 summarises the professional students’ scores on each of the five personality domains in comparison with approximately age-matched UK nursing students [46]. The professional students in this study had significantly higher mean neuroticism and openness to experience scores than the nursing students [46]. They also scored significantly lower on extraversion, agreeableness and conscientiousness.

**Table 2:** The mean scores of the professional students and UK nursing students on each of the five domains of personality measured by the NEO-FFI

Study	Sample	Personality Domain	Mean score	Professional students’ mean score	*p*-value*
Deary, Watson & Hogston (2003) [46]	124 UK nursing students	Neuroticism	21.6	24.67	*p* < 0.001
		Extraversion	32.0	27.44	*p* < 0.001
		Openness	26.6	28.87	*p* < 0.001
		Agreeableness	34.6	31.05	*p* < 0.001
		Conscientiousness	32.6	30.30	*p* < 0.001

*t-test *p*-value

### Perfectionism

Table 3 summarises FMPS scores overall and by population. After adjusting for age and gender, four of the six dimensions of perfectionism differed significantly by population. Pharmacy students scored significantly higher on the domains of ‘parental expectation’ (*p* = 0.001) and ‘parental criticism’ (*p* < 0.001) than the veterinary students (*p* = 0.002; *p* < 0.001). For ‘organisation’ (*p* = 0.002) dentistry students scored more highly than veterinary students (*p* = 0.007). Finally, on the dimension ‘concern over mistakes’ (*p* = 0.002) law students scored higher than the veterinary students (*p* = 0.002), medical students (*p* = 0.04) and dentistry students (*p* = 0.03). There was a significant difference among the populations in overall perfectionism scores (*p* = 0.001) with veterinary students scoring significantly lower than both pharmacy (*p* = 0.02) and law students (*p* = 0.005).

**Table 3:** Mean scores and standard deviations on each of the six dimensions of perfectionism, by population and overall

	Population					
	Veterinary ***N*** = 714	Medical ***N*** = 208	Pharmacy ***N*** = 133	Dentistry ***N*** = 145	Law ***N*** = 216	Overall ***N*** = 1416
	Mean (SD) 95% CI	Mean (SD) 95% CI	Mean (SD) 95% CI	Mean (SD) 95% CI	Mean (SD) 95% CI	Mean (SD) 95% CI
Parental Expectations	13.78 (4.49)* 13.45–14.11	14.29 (4.89) 13.62–14.92	15.73 (4.93)* 14.88–16.57	14.72 (4.58) 13.96–15.47	14.73 (4.89) 14.07–15.38	14.28 (4.70) 14.03–14.52
Parental Criticism	8.38 (3.37)* 8.13–8.62	8.88 (3.51) 8.40–9.36	9.92 (3.67)* ^†^ 9.29–10.55	8.63 (3.47) ^†^ 8.07–9.20	9.08 (3.83) 8.56–9.60	8.73 (3.54) 8.55–8.91
Organisation	21.33 (5.21)* 20.95–21.71	21.99 (4.77) 21.33–22.64	22.60 (4.60) 21.81–23.39	22.88 (4.96)* 22.06–23.69	21.64 (5.20) 20.94–22.34	21.75 (5.09) 21.49–22.02
Personal Standards	24.99 (4.87) 24.64–25.35	25.09 (4.89) 24.42–25.75	25.28 (5.06) 24.41–26.15	25.42 (4.80) 24.63–26.21	25.64 (4.58) 25.27–26.50	25.21 (4.84) 24.96–25.47
Concern over Mistakes	25.05 (7.52)* 24.50–25.60	24.94 (7.72)‡ 23.89–26.00	26.82 (7.78) 25.49–28.15	24.73 (7.27)^†^ 23.54–25.92	27.12 (7.65)*‡ ^†^ 26.09–28.15	25.48 (7.61) 25.09–25.88
Doubts about Actions	11.82 (3.40) 11.58–12.07	11.50 (3.23) 11.06–11.94	12.20 (3.24) 11.65–12.76	11.54 (3.08) 11.04–12.05	11.92 (3.52) 11.44–12.39	11.80 (3.35) 11.62–11.97
Overall MPS Scores	84.02 (17.61)* ^†^ 82.73–85.32	84.70 (17.80) 82.27–87.14	89.95 (18.45)* 86.78–93.11	85.05 (16.55) 82.33–87.76	88.73 (17.33) ^†^ 86.41–91.06	85.50 (17.68) 84.58–86.42

Scores marked with *, †, and ‡ represent significantly different pairwise comparisons

### Multivariable analyses

#### Wellbeing

The regression model for WEMWBS scores is shown in Table 4. Seven predictors accounted for 51% (R^2^ = 0.51) of the variance. Compared with being a veterinary student, being a law student, being a dentistry student, neuroticism and perfectionism were predictive of lower WEMWBS scores, indicating poorer levels of wellbeing. Being a pharmacy student, extraversion and conscientiousness were predictive of higher WEWMBS scores and thus more positive mental health. Neuroticism explained the greatest proportion of the variance.

**Table 4:** Multiple regression model for overall WEMWBS scores (*N* = 1405)

Variable	B^a^	SE B	β^b^	*p*-value
Constant	50.41	1.48		< 0.001
Population				
Veterinary	Ref	Ref	Ref	Ref
Medical	0.46	0.49	0.02	0.35
Pharmacy	0.13	0.59	0.004	0.03
Dentistry	−1.21	0.56	−0.04	0.03
Law	−1.29	0.48	−0.05	0.008
Neuroticism	−0.50	0.02	−0.51	< 0.001
Extraversion	0.29	0.03	0.23	< 0.001
Conscientiousness	0.10	0.02	0.08	< 0.001
Perfectionism	−0.03	0.01	−0.06	0.006

^a^Unstandardised coefficients, ^b^Standardised coefficients

#### Psychological distress

The GHQ-12 regression model is summarised in Table 5. Four variables explained 42% of the variance (R^2^ = 0.42). Compared with being a veterinary student, being a dentistry student, being a law student, and neuroticism all predicted increased GHQ-12 scores, which is indicative of heightened psychological distress. Extraversion, on the other hand, predicted lower GHQ-12 scores, denoting reduced levels of psychological distress. Neuroticism accounted for the greatest proportion of the variance.

**Table 5:** Multiple regression model for overall GHQ scores (*N* = 1406)

Variable	B^a^	SE B	β^b^	*p*-value
Constant	4.25	1.06		< 0.001
Population				
Veterinary	Ref	Ref		Ref
Medical	0.002	0.39	0.00	0.10
Pharmacy	0.39	0.47	0.02	0.40
Dentistry	0.97	0.45	0.05	0.03
Law	1.78	0.39	0.10	< 0.001
Neuroticism	0.39	0.02	0.55	< 0.001
Extraversion	−0.07	0.02	−0.07	0.003
Perfectionism	0.03	0.01	0.08	0.001

^a^Unstandardised coefficients, ^b^Standardised coefficients

#### Depression

The results of the BDI-II regression model are summarised in Table 6. Three variables predicted heightened BDI-II scores, indicating elevated levels of depression; these were being a law student compared with a veterinary student, neuroticism and perfectionism. Extraversion and conscientiousness predicted lower BDI-II scores, thus lower levels of depression. These predictors explained 52% (R^2^ = 0.52) of the variance in BDI-II scores. Neuroticism accounted for the greatest proportion of the variance.

**Table 6:** Multiple regression model for overall BDI-II scores (*N* = 1402)

Variable	B^a^	SE B	β^b^	*p*-value
Constant	−3.21	1.66		0.05
Population				
Veterinary	Ref	Ref	Ref	Ref
Medical	0.49	0.54	0.02	0.37
Pharmacy	1.33	0.66	0.04	0.05
Dentistry	1.05	0.63	0.03	0.10
Law	1.56	0.54	0.06	0.004
Neuroticism	0.57	0.03	0.51	< 0.001
Extraversion	−0.16	0.03	−0.11	< 0.001
Conscientiousness	−0.12	0.03	−0.09	< 0.001
Perfectionism	0.11	0.01	0.19	< 0.001

^a^Unstandardised coefficients, ^b^Standardised coefficients

#### Suicide attempts

The logistic regression model for suicide attempts is summarised in Table 7. Medical students were twice as likely to have attempted suicide as veterinary students. Increasing neuroticism, perfectionism and age were also associated with greater odds of suicide attempts. These predictors explained 19% (Negelkerke R^2^) of the variance.

**Table 7:** Logistic regression model for suicide attempts (*N* = 1366)

Variable	Odd Ratio (95% CI)	*p*-value
Population		
Veterinary	Ref	Ref
Medical	2.06 (1.11–3.84)	0.02
Pharmacy	1.61 (0.78–3.32)	0.20
Dentistry	0.98 (0.41–2.32)	0.97
Law	1.42 (0.74–2.71)	0.29
Neuroticism	1.08 (1.05–1.12)	< 0.001
Perfectionism	1.03 (1.02–1.05)	< 0.001
Age	1.10 (1.04–1.16)	0.001

The outcome variable is coded 0 = no suicide attempt, 1 = suicide attempt

## Discussion

This research aimed to investigate whether the big five personality traits and perfectionism varied significantly across the five student groups studied, and the association that this may have with mental ill-health in each population. Previous research has demonstrated a robust association between neuroticism and depression in particular [47, 48]. Using the NEO-FFI personality inventory, this study found that neuroticism was the most powerful predictor of psychological dysfunction, but that levels of neuroticism did not differ significantly among the five student groups. Furthermore, differences in morbidity remained significant after adjusting for neuroticism, indicating that other unmeasured factors are involved.

After adjusting for confounding by age and gender, law students scored significantly lower on agreeableness than the veterinary, dentistry and medical students. High agreeableness is characterised by altruism, trust in others, cooperation and empathy, whereas lower agreeableness is indicative of competitiveness, cynicism, scepticism and detachment [40]. Costa and McCrae [40] draw attention to the fact that low agreeableness does not signify a societal or psychological disadvantage. While it could be argued that empathy and cooperation, typified in high agreeableness, might attract an individual to train for, and be advantageous in, the medical occupations, these same traits might not necessarily confer an advantage in the law profession, which calls for detachment and objectivity.

Medical, veterinary and law students scored significantly higher on openness to experience than dentistry and pharmacy students. Conflicting results have been obtained from different studies with some identifying associations between openness and wellbeing [49], others identifying associations between openness and psychological disorders [15, 50] and others no relationships at all [51]. These contradictory findings could reflect the suggestion that open individuals experience both positive and negative emotions more keenly than less open people [40]. In the current study, although medical, veterinary and law students scored highly on openness, this domain was only predictive of depression and psychological distress for law students. Openness is typified by imagination, intellectual curiosity, attentiveness to inner feelings and a need to magnify and analyse experiences [50, 52]. It is possible that openness perpetuates already existing depression rather than being a causal factor in its development. Therefore, where depression is already present, openness may predispose greater inward focus upon emotions and negative interpretation of feelings, which are central features of the cognitive theory of depression [53].

Mean scores indicated that medical students showed the highest levels of extraversion and pharmacy students the lowest. However, while there was weak evidence for an overall significant variation in extraversion by student group, post-hoc analyses did not detect significant pairwise differences. Previous research has shown that compared to introverts, extraverts have larger social networks, solicit social support more readily and feel a greater sense of belonging to their supportive network [54]. The more solitary nature of the law degree potentially limits opportunities for being sociable and forming supportive group affiliations, and introverted individuals could be attracted to a degree course which emphasises independent working.

The last of the big five traits assessed was conscientiousness. This trait has been inversely associated with suicidal ideation and psychiatric disorders [15, 22]. It is also positively associated with more adaptive coping [23]. Conscientiousness did not differ significantly across student groups in this study. This is not surprising, as many conscientious characteristics, such as organisation, thoroughness and self-discipline would be valuable in pursuing any of these professional degrees. However, when compared with a population of UK nursing students [46], the students in this study scored significantly lower for conscientiousness. This is could be owing to the relatively young age of the participants, as conscientiousness has reliably been shown to increase with age [55–57]. Costa and McCrae [58] demonstrated that, compared with older adults, college students had consistently lower scores on this domain. However, the mean age of the nursing students [46] was similar to that of the professional students in this study (25 years compared with 22 years), which suggests that age does not fully explain this difference. Lack of other suitably age-matched data from non-clinical populations in the UK, meant that we could not make any further meaningful inter-population comparisons.

In this study, after adjusting for age and gender, perfectionism scores varied significantly across groups, with veterinary students scoring the lowest of all groups and significantly lower than both pharmacy and law students. Regression analyses indicated that perfectionism was associated with poorer psychological health and wellbeing, and increased likelihood of suicide attempts, which is consistent with existing evidence [26, 30]. Although it is a popular and plausible anecdotal view that professional students are excessively perfectionistic, this is not supported by available evidence and there is little empirical research into this trait in UK student groups. One US study that compared perfectionism among medical, dentistry, nursing and pharmacy students using Hewitt and Flett’s Multidimensional Perfectionism Scale [32] did not detect differences in perfectionism between the healthcare students and the general student population [27]. However, it was shown that those with heightened perfectionism were at greater risk for psychological distress. Our findings that perfectionism was a risk factor for mental ill-health corroborate this.

It is suggested that personal standards and organisation are adaptive facets of perfectionism, while doubts about actions, concern over mistakes, parental criticism and parental expectations are maladaptive [59]. Veterinary students in our study scored significantly lower on the perception of high parental expectations and criticism than the pharmacy students, suggesting that their motivation for pursuing the veterinary degree could be intrinsic and not driven by external factors, while the pharmacy students might have been more influenced by parental pressure. Intrinsic motivation to achieve a goal is associated with greater wellbeing, whereas the achievement of extrinsically imposed goals does not confer such benefits [60, 61]. It follows that pursuing a professional course because of extrinsic reasons, for example parental expectations, is likely to be less wellbeing enhancing than if the motivation comes from within the individual and an interest in the degree course for itself.

Overall, the professional students in this study had higher levels of neuroticism and lower conscientiousness than a UK nursing population [46], potentially reflecting the relatively young age of this sample, but were also agreeable and open to experience. The comparisons that can be made, and conclusions drawn, are restricted by the limited number of published studies using the same measures with similar populations to investigate personality and perfectionism. Within the student groups surveyed, law and pharmacy students had the lowest agreeableness scores, and the highest levels of perfectionism. These two populations are also the most neglected by researchers, certainly in the UK. While it is a popular opinion that professional students may be competitive, ‘Type-A’ individuals afflicted with excessive neuroticism and perfectionism, there is currently not enough evidence to substantiate these assertions. In order to verify whether this indeed is the case, robust empirical research using validated measures need to be conducted rather than relying upon anecdotes, which risk becoming taken-for-granted truths.

A limitation of this study is the unequal proportion of responses from each of the populations. While large numbers of veterinary medicine students participated, there were far fewer responses from medical students. This may be in part because of differences in recruitment methods. Veterinary students were contacted directly by the researcher, and pharmacy, law and dentistry students were emailed by a third party within each institution. However, the study was only advertised on e-message boards for the medical students, following institution protocols. This has implications for the generalisability of results for the groups with fewer responses. The extent to which these results may be generalised are further limited by the fact that only one veterinary and one pharmacy school participated in the study. However, no differences were identified between the different dentistry, medical and law schools who participated, which implies that these are continuous populations. It cannot be assumed that similar results would be identified in other pharmacy and veterinary populations, but future comparative research can build upon these initial findings.

## Conclusions

This study identified that veterinary students had the lowest perfectionism scores of the five student groups surveyed. They also scored more highly on the trait of agreeableness than law students. Concurring with previous literature, high levels of neuroticism and low conscientiousness were risk factors for increased psychological morbidity in each of the student populations surveyed. These findings suggest that the prevailing anecdotal view of professional students possessing maladaptive personality traits that negatively impact on their mental health may be misplaced.

## Data Availability

The datasets used and/or analysed during the current study are available from the corresponding author on reasonable request.

## Abbreviations

NEO-FFI: NEO Five Factor Inventory
FMPS: Frost Multidimensional Perfectionism Scale
WEMWBS: Warwick-Edinburgh Mental Well-being Scale
GHQ-12: General Health Questionnaire-12
BDI-II: Beck Depression Inventory-II
